# Hommage to Richard R. Ernst

**DOI:** 10.3389/fmolb.2021.769772

**Published:** 2021-10-21

**Authors:** Anja Böckmann, Rachel Martin, Ann E. McDermott, Beat H. Meier, Annalisa Pastore, Erik R. P. Zuiderweg

**Affiliations:** ^1^ Molecular Microbiology and Structural Biochemistry, UMR 5086 CNRS, Lyon University, Lyon, France; ^2^ Department of Chemistry, University of California, Irvine, Irvine, CA, United States; ^3^ Department of Molecular Biology and Biochemistry, University of California, Irvine, Irvine, CA, United States; ^4^ Department of Chemistry, Columbia University, New York, NY, United States; ^5^ Laboratory of Physical Chemistry, ETH Zurich, Zurich, Switzerland; ^6^ King’s College London, London, United Kingdom; ^7^ Department of Biological Chemistry, University of Michigan Medical School, Ann Arbor, MI, United States; ^8^ Faculty of Science, Radboud University, Nijmegen, Netherlands

**Keywords:** NMR, solid, solution, method, applications

## Introduction

Richard R. Ernst (1933–2021) developed key tools that are the basis of today’s applications in the field of Molecular Bioscience: Fourier transform spectroscopy, multidimensional NMR, Fourier imaging to name the most important ones, but also many important technical improvements in solution- and solid-state NMR as well as new theoretical concepts.

Richard studied Chemistry at the ETH Zurich and got his PhD 1962. He was then a researcher at Varian Associates in Palo Alto. In 1968 he returned to the ETH Zurich, first as a lecturer, then as an assistant professor and from 1976 as a full professor in Physical Chemistry until his retirement in 1998. In California he developed, together with Weston Anderson, Fourier transform NMR, which not only yielded an important sensitivity enhancement, but was the basis of the multidimensional methods which he developed, following his return to ETH Zurich, based on ideas of Jean Jeener. This methodology formed the foundation for many groundbreaking advances in magnetic resonance, and notably in its applications to biology and medicine. In a fruitful collaboration with Kurt Wüthrich, the first multidimensional protein spectra were obtained laying the foundation for structural biology applications. Besides these main developments, Richard’s research group at ETH has led many developments in NMR spectroscopy, both in solution and in solids. These advances have made NMR spectroscopy a central analytical method for almost all chemists biochemists. An important application area is structural biology, and his work prepared NMR spectroscopy to become, together with X-ray crystallography and electron microscopy, one of the pillars of protein structure determination.

Richard Ernst has received many honors and awards for his scientific work, including the Ampere Prize (1990), the Wolf Prize in Chemistry (1991), the Horwitz Prize in Biology and Biochemistry (1991), and the Nobel Prize in Chemistry (1991). Richard was a highly creative researcher, and of great influence in his field and beyond. He was an excellent teacher and an inspiring member of the NMR community.

With the purpose of remembering Prof. Richard R. Ernst as one of the great contributors to molecular biosciences, some of the editors of Frontiers in Molecular Biosciences contributed informal messages to this short collection.

## Rachel Martin

For someone who started graduate school, as I did, in the early days of biomolecular solid-state NMR, it is literally impossible to imagine the field without the contributions of Richard Ernst. His groundbreaking work directly enabled protein structure determination, as well as many other ubiquitous applications in chemistry. However, my most lasting memories are of interacting with him at conferences, where he was a warm and enthusiastic member of our community. Prof. Ernst had already been a Nobel laureate for several years by the time I first met him at my first ENC, but he was very kind and encouraging. He always made time to talk with young people, and not only about science, but about living well and doing good. Beyond NMR, he taught me about the importance of engaging with political issues as well as seeking out and appreciating art and music. I admired his wide range of interests and encyclopedic knowledge, from classical music, to Tibetan art and culture, and of course, his enthusiasm for exploring our world with all of the tools we can develop. We are lucky to have had him in the NMR community, as a researcher, a leader, and a friend.

## Ann E. McDermott

A teacher, a scientist lives by clarity of mind. This phrase describes no one better than Richard Ernst. Richard is honored for his many revolutionary accomplishments and contributions to the toolkit of NMR and all of spectroscopy, including the first examples of FT-NMR and multidimensional spectroscopy. I knew and remember Richard also for his lectures, which were a beautifully-paced walk through the most important aspects of NMR in startling clarity. I was lucky to be hanging on every word as he delivered a series of lectures to the Harvard MIT community in the late ‘80s, and I was transitioning from X-ray absorption spectroscopy in the labs of Ken Sauer and Mel Klein at Berkeley, to NMR as a newly minted postdoc in the laboratory of Bob Griffin. I was utterly unaware that others might have felt his lectures to exceed the normative 60 min; for me there was no clearer nor more inspiring time in my week, and I would have literally stayed as long as he was speaking. I thought of the points he made many times as I crawled my way through various NMR tomes; his remarks provided for me a kind of roadmap of what is possible when systems are well behaved.

As outstanding a teacher as he was, it is possible that there could at times be too much generosity and too much clarity. In 1991, I was humbled and out of my depth by many aspects of my new role on the faculty at Columbia University, and particularly so to be one of the hosts for both Kurt Wüthrich and Richard Ernst as they were joint recipients of one the highest honors Columbia University awards, the Louisa Gross Horwitz Prize. I was surprised during that visit that he recalled some of the questions and discussions we had had back in Boston, that he indicated his support for a young woman entering this field of science, and that he took the time to offer thoughtful and useful feedback on the program I wanted to develop. The atmosphere of this visit was much affected by the fact that en route he learned of his Nobel prize. Despite the “old school” formality to his bearing, he had his own way of making others comfortable in his presence and focusing on things that are truly important in his conversations, and he shared with me his deep discomfort in receiving this prize, in the light of the contributions of Jean Jeener, Kurt Wüthrich, and others. Many who knew him better have remarked that Richard had profound humility; I certainly experienced that. During this visit, Richard was again generous with his time as a lecturer. In our beloved 309 Havemeyer classroom, now the Gilbert Stork Lecture Hall, he filled the room with almost 300 chemists, biochemists, and physicists. He and I and our then director of undergraduate education, Leonard Fine, had spent our lunch hour organizing and loading three full carousels of slides alongside two fat 3-ring notebooks full of transparencies for the afternoon’s nominally 50 min lecture. Richard’s exuberant leadership style signaled to me that I did not need to think too deeply about how such a vast collection of media might play out. At 6 pm, more than 70 min into his lecture, with surprisingly few people leaving to check their reactions or collect kids from daycare, it was revealed that the second carousel had been loaded upside-down. Richard’s evident displeasure, and his assumption that I was responsible for this calamity, had a wonderful result. The excellent theory group in our department at the time, and the organic chemistry heavyweights at Columbia, all leaned slightly forward from their seats in the front row to give me a collective big thumbs up. It was the clearest sign I could imagine that I was doing ok in my new role as professor. Richard continued to support me and so many other people junior to him in magnetic resonance, in more orthodox and very substantial ways, throughout his life. I thank the editors for providing this opportunity to join many NMR spectroscopists in praising and sharing gratitude for his brilliance, clarity and generosity.

## Beat H. Meier

I had the privilege to spend, between 1978 and 1994, over a dozen years in Richard Ernst’s research group as a master student, graduate student, postdoc and senior scientist. It was an exciting and productive time. Richard had created an environment, intellectually and hardware-wise, that was unique. This was not easy. Richard was hired, as he describes in his autobiography, as an assistant with the expectation to run good spectra for the chemists—which was close to impossible because the old equipment and the magnetic-field fluctuations generated by the Zurich streetcars passing in front of the institute ruined most experiments except from half past midnight till four in the morning. The development from there to a leading lab took hard work and persistence. When I entered the lab in 1978, 2D spectroscopy was in full development and the home-build hardware was already available though still primitive. The “fast” 2D Fourier transform took a quarter of an hour. Working with Richard was easy, he gave the students a lot of scientific freedom, still closely following what we were doing. Richard was very demanding, especially of himself, and had a fine sense for challenging his coworkers with difficult problems, but only asking as much as was within their personal capabilities. Most importantly, Richards was a constant source of interesting suggestions and critical questions. His interests were incredibly broad covering solid-state NMR, solution-state NMR, liquid-crystalline systems and imaging. In Richards lab I was, several times, involved in industrial collaborations as the commercial production of advanced NMR instruments was always of interest for Richard.

## Annalisa Pastore

I joined Ernst’s lab in 1984–1985 with a fellowship between ETH and the Italian Ministry. The laboratory was downtown Zurich, in the department of Physical Chemistry. It was also a special time for the group. The paper by Sørensen et al. (1984) on the product operator formalism had been published only a year earlier. The lab comprised a unique group of mythical people who included Geoffrey Bodenhausen, Malcolm Levitt, Dieter Suter and many others. Ernst and Bodenhausen were writing The book “Principles of NMR in 1 and 2D.” The atmosphere was international, challenging, vibrant.

I was over the moon for being there and felt in the Olympus of NMR. But it was not always easy. Just to give an idea of what ETH was at the time: I was the first woman in Ernst’s group and in the whole there were only three women: Ernst’s secretary, a PhD student and myself. ETH got its first ordinary female professor, with the appointment of architect Flora Ruchat-Roncati, in 1985…

I remember fondly Ernst’s lectures on measure theory. They were magnificent. The group at the time was purely working on the technique. The word biology had not yet entered the lab. I worked on a pulse sequence (i.e., a scheme of radiofrequency pulses) called ZZ exchange spectroscopy. It allowed to measure the kinetics of interconversion between different molecular states. Now this sequence has been applied to detect differences between folded and unfolded states, or ligand-free and ligand-bound forms of a protein. At the time we tried to implement it on simpler systems.

It was interesting to see how Ernst worked. He had full freedom of research. He could get an idea perhaps from the last conference attended. And then he would put a bright PhD student on the problem. This is what happened with 2D NMR and, when I was in the lab, with zero-field NMR. This working model would be unthinkable now at least in the United Kingdom where also permanent positions can be interrupted in a flash and where we are continuously asked to work on translational targets.

On the whole I think Richard was a great scientist, of a type that seems to become extinct.

## Erik R. P. Zuiderweg

I took classes 2D NMR from Professor Ernst at the ETH Zurich in 1983. He was a very gifted teacher, besides, as we all know, a scientific giant.

At that time, he was already famous, but remained very humble and approachable as a human being. And he remained humble and approachable after his very well-deserved Nobel Prize.

With his collaborations with Professor Kurt Wüthrich and Dr. Gerhard Wagner, Ernst contributed much to the founding of solution NMR as a major technique in structural biology.

The key experiment is the “Two-dimensional nuclear Overhauser enhancement (2D NOE) experiment for the elucidation of complete proton-proton cross-relaxation networks in biological macromolecules” which appeared in BBRC (sic!) in 1980. The title of the paper says it all: if you can measure (and interpret) the complete proton-proton cross relaxation network, you have the (dynamically averaged) structure in hand. This experiment has later been extended into additional dimensions, and still is indispensable for structure determination. To date more than 13,000 “NMR structures” have been deposited in the PDB.

## Anja Böckmann

I never really met Richard Ernst. Still, I met his work—for the first time when I was doing my physical chemistry courses in 1991 at the TU Berlin. I still remember the seminar at the Institute where his winning of the Nobel Prize was discussed. Some months later I decided to go into NMR, with Dieter Ziessow, who was then a Professor in Physical Chemistry at TU Berlin. In the end I do not remember what drove me there, but it were Dieter’s lively lessons on spin operators, using the product operator formalism introduced by Richard Ernst and coworkers, which made me addicted, and directed my future career towards the field. Richard Ernst also made important contributions to structural biology, even if this might not be what others most remember - his presenting of the first solid-state NMR sequential assignments of Ubiquitin done by Suzana Straus at the EUROMAR/ISMAR in Berlin left a lasting impression on me. I met Richard Ernst’s work again later at ETH Zurich, during my collaboration with Beat Meier, where I deeply admired the laboratory culture the former had so successfully established, and the latter not less successfully driven to the current state of the art and beyond.
